# Pitfalls and solutions of the fully-automated radiosynthesis of [^11^C]metoclopramide

**DOI:** 10.1186/s41181-019-0083-2

**Published:** 2019-12-18

**Authors:** Verena Pichler, Marius Ozenil, Karsten Bamminger, Chrysoula Vraka, Marcus Hacker, Oliver Langer, Wolfgang Wadsak

**Affiliations:** 1 Division of Nuclear Medicine, Department of Biomedical Imaging and Image-guided Therapy, Medical University of Vienna, Vienna, Austria; 2 Center for Biomarker Research in Medicine, CBmed GmbH, Graz, Austria; 3 Preclinical Molecular Imaging, AIT Austrian Institute of Technology GmbH, Seibersdorf, Austria; 4 Department of Clinical Pharmacology, Medical University of Vienna, Vienna, Austria

**Keywords:** [^11^C]metoclopramide, carbon-11, radiosynthesis, blood-brain-barrier, PET

## Abstract

**Background:**

[^11^C]Metoclopramide is a new radiotracer for investigating the activity of P-glycoprotein at the blood-brain barrier. A highly stable and reproducible radiosynthesis is a prerequisite for clinical studies applying [^11^C]metoclopramide or other ^11^C-labelled radiotracers, therefore all potential pitfalls must be identified and monitored to allow a stable process.

**Results:**

Long-term production (*n* = 94 in a time range of approximately 2 years) of [^11^C]metoclopramide synthesized on two commercially available synthesizers yielded 3.9 ± 2.0 GBq of product with a molar activity of 132 ± 164 GBq/μmol and an overall success rate of 93%. During all successful productions, the product quality was in accordance with the recommendations of the European Pharmacopoeia. The most common pitfalls that were identified for the radiosynthesis included poor turnover into [^11^C]CH_3_OTf, decomposition of the solvent or insufficient semi-preparative HPLC performance.

**Conclusion:**

The study provides long-term insight in the improved, robust and stable preparation of [^11^C]metoclopramide for human use.

## Background

[^11^C]Metoclopramide is a novel positron emission tomography (PET) radiotracer, which has been developed to measure the activity of P-glycoprotein (P-gp) at the blood-brain-barrier, an important efflux transporter, which restricts the brain distribution of many different drugs (Bauer et al. 2019; Tournier et al. 2019; Auvity et al. 2018; Pottier et al. 2016). [^11^C]Metoclopramide is a weak P-gp substrate, which has considerably higher brain uptake under conditions of full P-gp function as compared with previously described avid P-gp substrates for PET (e.g. [^11^C]verapamil or [^11^C]*N*-desmethyl-loperamide). Consequently, [^11^C] metoclopramide may possess a better sensitivity to measure moderate changes in P-gp function at the blood-brain barrier than previously described radiotracers (Bauer et al. 2019). Carbon-11 is an attractive radionuclide for PET imaging as it allows to radiolabel most drug molecules without structural changes. Moreover, its short radioactive half-life (20.4 min) leads to relatively low radiation exposure of study participants and allows for repeated PET scanning within one imaging session (e.g. two consecutive scans without and with pre-treatment with a P-gp inhibitor). For a successful clinical application, stable and reliable radiosynthesis procedures are therefore a prerequisite.

**Fig. 1:**
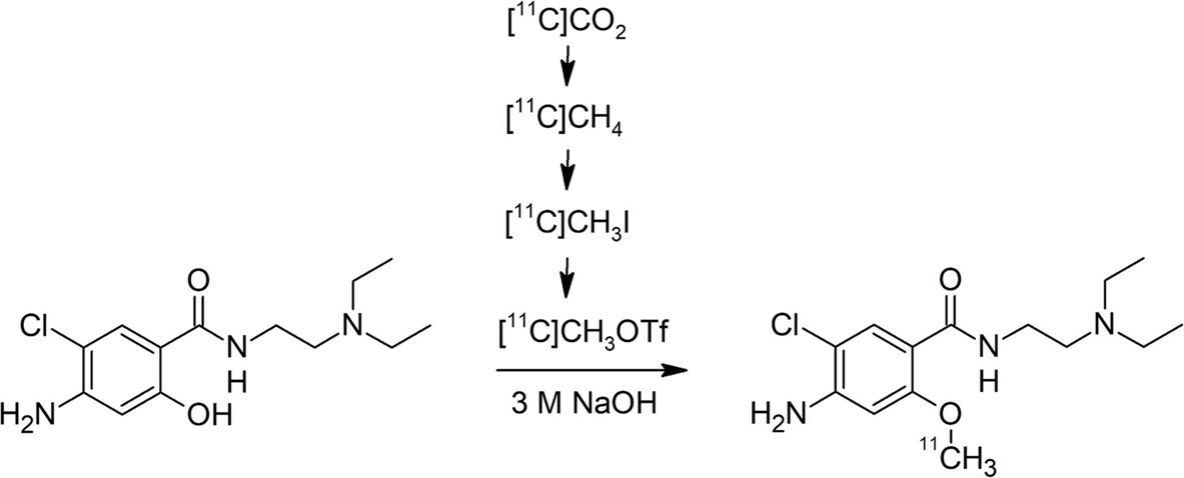
Scheme for the radiosynthesis of [^11^C]metoclopramide

Here, we present the fully-automated radiosynthesis of [^11^C]metoclopramide (Fig. 1) conducted with a GE TRACERlab™ FX C Pro or GE TRACERlab™ FX2 C system, respectively. The main pitfalls of the radiosynthesis were identified e.g. decomposition of solvent or insufficient separation on semi-preparative HPLC and appropriate solutions were implemented.

## Material and methods

All chemicals were obtained from commercial sources and used as received. The precursor *O*-desmethyl-metoclopramide (GMP grade) as well as the reference compound metoclopramide (4-amino-5-chloro-*N*-[2-(diethylamino)ethyl]-2-methoxy-benzamide) were purchased from ABX (Radeberg, Germany). The Ni catalyst (Shimalilte Ni reduced, 80/100 mesh) was purchased from Shimadzu (Kyoto, Japan). SepPak® C18 plus cartridges for solid-phase extraction were purchased from Waters (Waters® Associates Milford, MA, USA). Low-protein binding Millex GS® 0.22 μm sterile filters were purchased from Millipore® (Bedford, MA, USA). The silver triflate column was prepared according to the recommendation of the module producer General Electric Medical System, Uppsala, Sweden. Silver triflate and carbopack adsorbent C, 80–100 mesh was obtained by Sigma Aldrich and used as received.

### Instrumentation

[^11^C]CO_2_ was produced in a GE PETtrace 860 cyclotron (General Electric Medical System, Uppsala, Sweden) via the ^14^N(p,α)^11^C nuclear reaction by irradiation of a gas target (aluminium) filled with N_2_ + 1% O_2_ (Air Liquide, Vienna, Austria). Typical beam currents were 60–65 μA and the irradiation was stopped as soon as the desired activity level was reached (approx. 80–130 GBq [^11^C]CO_2_; corresponding to 30–40 min irradiation time). The synthesis of [^11^C]metoclopramide starting from [^11^C]CO_2_ (Fig. 1) was performed in two different GE modules (GE Healthcare, Uppsala, Sweden), namely a TRACERlab™ FX C Pro and a TRACERlab™ FX2 C module. For analytical HPLC, an Agilent 1260 system was used, which was equipped with a quaternary pump (G1311B), a multi wavelength UV-detector (G1365D), a column oven (G1316A), a manual injector (G1328C), as well as a radio-detector controlled by GINA Star software (Elysia-Raytest; Straubenhardt, Germany). Osmolality of the formulated radiotracer solution was measured using a Wescor osmometer Vapro® 5600 (Sanova Medical Systems, Vienna, Austria) and pH was always measured using a WTW inoLab 740 pH meter (WTW, Weilheim, Germany) for every released product. Gas chromatography was performed using a 430-GC system (Bruker Daltonik GmbH, Bremen, Germany) and a capillary column (forte GC Capillary Column ID-BP20; 12 m × 0.22 mm × 0.25 μm) from SGE Analytical Sciences Pty Ltd. (Victoria, Australia). Butanone was analysed with a Bruker Avance III 200 spectrometer (200 MHz for ^1^H).

### Preparation of the synthesizers

**Fig. 2:**
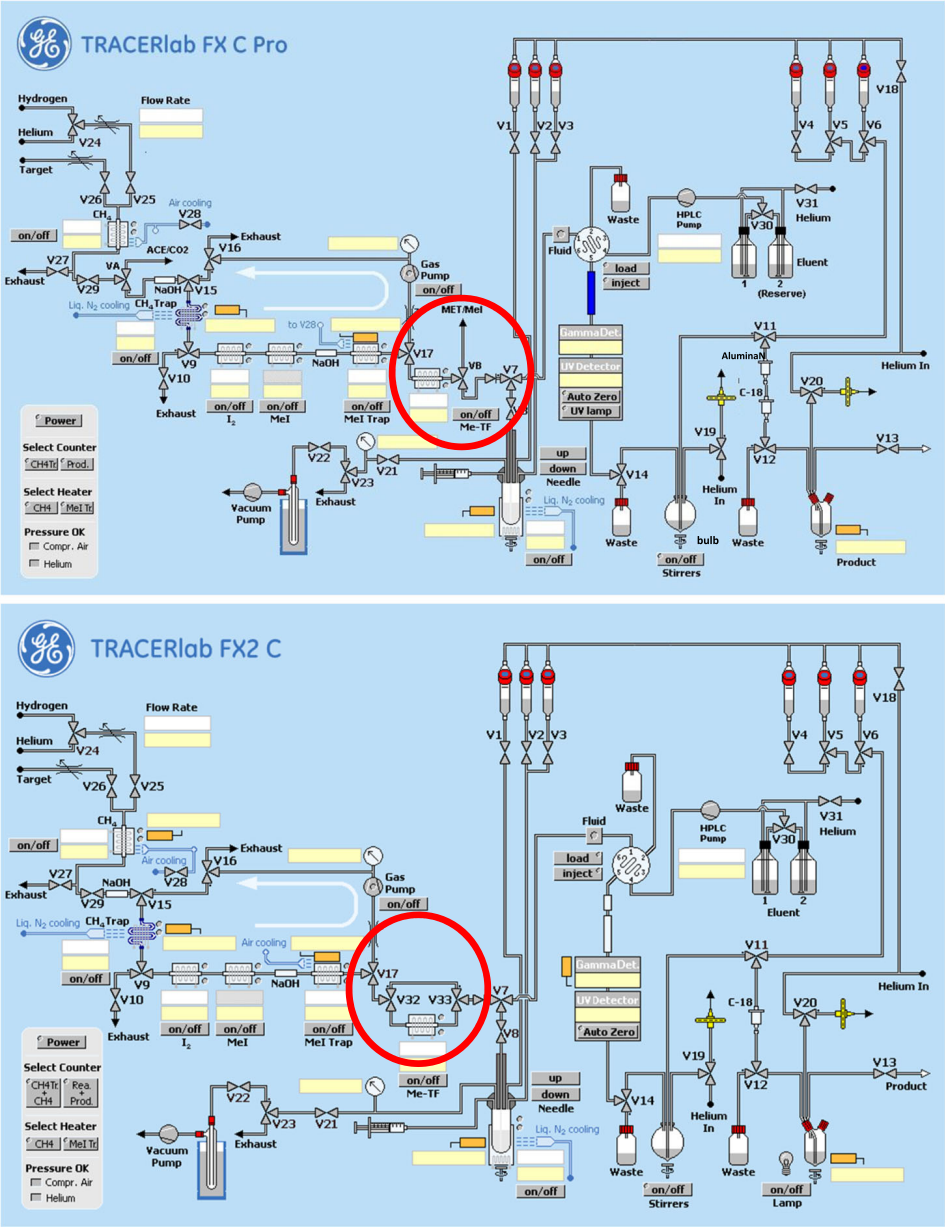
Flow charts of the used synthesis modules TRACERlab™ FX C Pro and TRACERlab™ FX2 C. The major differences between the set-ups concern the installation of the silver triflate column are highlighted (red circle)

The flow charts of the TRACERlab™ FX C Pro and FX2 C are depicted in Fig. 2. All parts involved in the radiosynthesis were first flushed with helium and subsequently checked for tightness by applying a helium flow of 100 mL/min, which has to decrease below 4 mL/min when all lines are tightly sealed. The methyl iodide trap was heated to 200 °C for approximately 20 min and afterwards cooled down to ≤35 °C. The silver triflate column was preheated to 200 °C before start of the synthesis for approximately 15 min. The reaction vessels V1-V3 and the corresponding transfer lines via the reaction vial and HPLC injection loop into the waste were washed twice with water and acetone. Complete removal of all traces of solvent was assured by visual inspection of all connected tubes. The radiotracer formulation part of the module was washed with water and ethanol including the bulb and product collection vial. The preparation procedure was performed similarly for the GE TRACERlab™ FX C Pro and TRACERlab™ FX2 C. A detailed protocol for the module preparation was published recently (Vraka et al. 2019).

The reaction vials were filled with 1 mL water (aqua ad injectabilia; V2), 5 mL sodium chloride 9% (V4), 1.5 mL ethanol (V5) and 10 mL water (aqua ad injectabilia; V6). The bulb was filled with 80 mL of water (aqua ad injectabilia), a C18 Plus Sep-Pak (equilibrated with 10 mL ethanol and 20 mL water) was placed on the Sep-Pak position and 6 mL of phosphate buffered saline (prepared by Vienna General Hospital pharmacy, Austria; 0.021 M phosphate buffer, 0.188 M NaCl, pH 7.7) were placed in the product collection vial. For patient application, a product vial with sterile filter and sterile air ventilation was prepared in a laminar air flow cell and connected to the product tubing. Prior to use, the reaction vessel had been stored at 100 °C for at least 4 h. Immediately before start of the radiosynthesis, 1 mg of *O*-desmethyl-metoclopramide (3.5 μmol) was dissolved in 400 μL butanone and transferred to the reaction vessel. Then 7 μL of 3 M aqueous NaOH solution (21 μmol, 6 equivalents) was added to deprotonate the phenol.

### Fully-automated radiosynthesis of [^11^C]metoclopramide

The synthesis sequence started with a 15–20 min pre-run including heating of the carbon dioxide trap to 400 °C and subsequent cooling to < 50 °C. During the heating process, the trap was continuously flushed with helium (50 mL/min). When 400 °C was reached, 3 min of hydrogen pre-equilibration of the carbon dioxide trap took place. Afterwards the carbon dioxide trap was cooled to < 50 °C and the methane trap was cooled to − 75 °C ending the pre-run with the comment ‘ready to receive activity’.

**Fig. 3:**
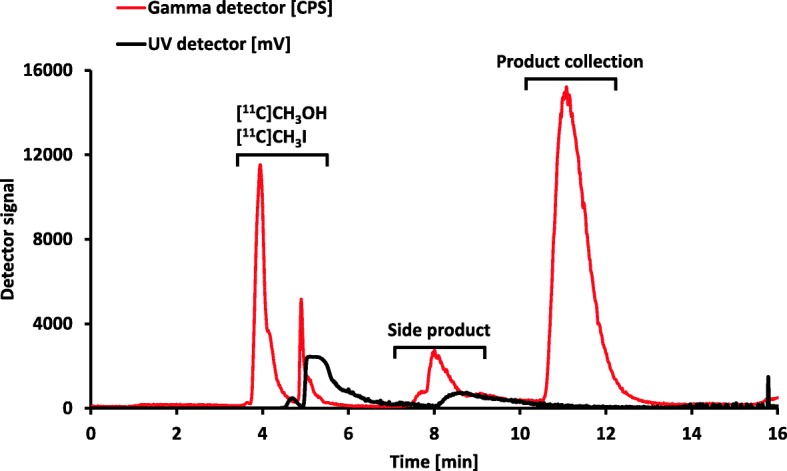
Typical semi-preparative RP-HPLC chromatogram of a [^11^C]metoclopramide radiosynthesis including the signal of the UV-detector (254 nm, black) and radio-detector (red). The product peak has a retention time of approximately 10.5–12.5 min

The automatic radiosynthesis was then started by delivering 116 ± 17 GBq of [^11^C]CO_2_ to the carbon dioxide trap and confirmation of the end of delivery. The gas phase transformation of [^11^C]CO_2_ via the formation of [^11^C]CH_4_ produced approximately 50 GBq of [^11^C]CH_3_I and has been described in detail elsewhere (Vraka et al. 2019; Pichler et al. 2018a; Philippe et al. 2018; Larsen et al. 1997). [^11^C]CH_3_OTf was produced by passing [^11^C]CH_3_I gas through the silver triflate column at 200 °C. After completion of the activity trapping within the reaction mixture, the reaction took place at 110 °C for 2 min. Afterwards the reaction mixture was quenched with water from V2 and transferred to the semi-preparative HPLC via the injection valve. For semi-preparative HPLC, a SUPELCOSIL LC-ABZ+ column (5 μm, 250 mm × 10 mm) from Merck (Darmstadt, Germany) was eluted with freshly prepared 20 mM aqueous NaH_2_PO_4_ / acetonitrile (80/20) at a flow rate of 5 mL/min with UV absorption measured at 254 nm (Fig. 3).

In total, 70 syntheses were performed on the TRACERlab™ FX C Pro, of which the first eight syntheses were performed for establishment and evaluation of the automated radiosynthesis and analytical and preparative HPLC establishment. These syntheses were excluded from statistical analysis as the final activity yield was not always measured. Three syntheses failed, caused by insufficient transfer of the activity into the reactor (no formation of [^11^C]CH_3_I or [^11^C]CH_3_OTf), insufficient separation or no product formation at all besides other technical issues. Thirty-eight radiosyntheses were performed so far on the GE TRACERlab™ FX2 C, of which six syntheses were performed for validation of the new module (excluded data) and four synthesis failed. Out of these productions (*n* = 94, excluding test syntheses, 7 fail synthesis), 81 radiosynthesis were released for clinical application, the remaining six productions were released but for other applications. Details on optimization radiosyntheses performed, which were not released for human application but are included in the overall analysis are shown in Table 1. The success rate of [^11^C]metoclopramide produced on TRACERlab™ FX C Pro or GE TRACERlab™ FX2 C was 95 and 88%, respectively. The productions were performed between August 2017 and September 2019. The change of the synthesis module was implemented in December 2018.

**Table 1:** Overview on evaluation batches and overall yield of [^11^C]metoclopramide productions with changing reaction conditions

Solvent	Reactive synthon	RCY (% based on [^11^C]CO_2_, decay corrected)	Activity yield [GBq]
Acetone (*n* = 3)	[^11^C]CH_3_OTf	20.7 ± 4.5%	5.4 ± 1.2
Butanone (*n* = 3, evaluation batches)	[^11^C]CH_3_OTf	20.8 ± 3.8%	6.3 ± 1.7
Butanone (*n* = 1)	[^11^C]CH_3_I	no product	0
Butanone (*n* = 94, overall productions)	[^11^C]CH_3_OTf	13.2 ± 6.7	3.9 ± 2.0

### Quality control of [^11^C]metoclopramide

**Fig. 4:**
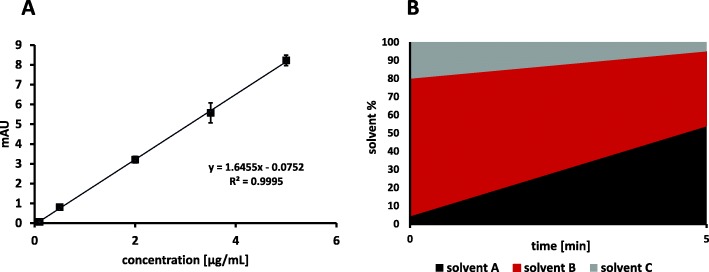
**a** Calibration curve of metoclopramide in the range of 0.1–5 μg/mL measured at a wavelength of 225 nm. The limit of detection and limit of quantification were 0.2 μg/mL and 0.7 μg/mL, respectively. **b** Gradient for the analytical RP-HPLC for the quality control of [^11^C]metoclopramide. The mobile phase consist of solvent A: aqueous acetonitrile (90%), solvent B: water and solvent C: aqueous ammonium phosphate buffer (50 mM), pH 9.3 adjusted with NaOH

**Fig. 5:**
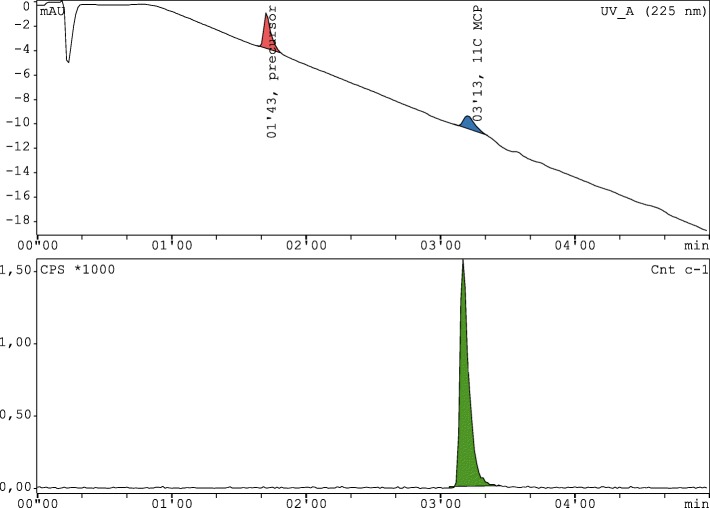
Exemplary analytical RP-HPLC chromatogram of [^11^C]metoclopramide formulated for i.v. injection including the signal of the UV-detector (225 nm, upper channel) and radio-detector (lower channel)

The quality control parameters encompassed the parameters pH, osmolality, organic solvent content, radionuclidic purity, radiochemical and chemical purity as well as biological testing of sterility and endotoxins. All tests performed were in accordance with the recommendations of the European Pharmacopoeia for other ^11^C-radiotracers. The chemical and radiochemical purity was measured by means of RP-HPLC using an X-Bridge BEH Shield RP-18, 4.6 × 50 mm, 2.5 μm, 130 Å (Waters GmbH) column eluted at a flow rate of 1 mL/min with a gradient program (Fig. 4) of the following solvents: solvent A: aqueous acetonitrile (90%), solvent B: water and solvent C: aqueous ammonium phosphate buffer (50 mM), pH 9.3 adjusted with NaOH (Nics et al. 2018). The retention time of the precursor *O*-desmethyl-metoclopramide was between 1 min 20 s to 1 min 50 s and for the product [^11^C]metoclopramide 2 min 50 s to 3 min 20 s (Fig. 5). The calibration curve was set up at 225 nm for the concentration range 0.1–5 μg/mL (0.1, 0.5, 2, 3.5 and 5 μg/mL standards) including blank measurements all performed in at least three repetitions (Fig. 4).

The retention times were checked prior every radiosynthesis by injection of the respective reference compounds. Both, the semi-preparative as well as the analytical HPLC systems were tested for the retention times of the reactive intermediates methyl iodide, methyl triflate and methanol (from the reaction of methyl triflate with water) as well as [^11^C]CH_3_I, [^11^C]CH_3_OTf, [^11^C]CO_2_ and a mixture of [^11^C]CH_3_OTf with water (for the retention time of [^11^C]CH_3_OH).

### Statistical analysis

Yield calculation consisted of the measured activity of the product in GBq corrected for decay divided by the produced starting activity of [^11^C]CO_2_ in GBq and is expressed in % if not stated otherwise (Coenen et al. 2017).

If not indicated otherwise, all values are depicted as mean ± standard deviation. All results derived from synthesis or quality control were analysed by means of MS Excel® 2013. Significance was tested with GraphPad Prims 6 by means of t test analysis.

## Results and discussion

### Automated radiosynthesis optimization for human application

Starting from 116 ± 17 GBq of [^11^C]CO_2_, 39 ± 13 GBq of [^11^C]CH_3_OTf was trapped in 400 μL butanone within the reactor containing *O*-desmethyl-metoclopramide and aqueous sodium hydroxide solution after approximately 15 min. In the original radiosynthesis published by F. Caillé and coworkers (Caillé et al. 2018) the [^11^C]CH_3_OTf was trapped at − 20 °C in acetone, with subsequent heating to 110 °C. This radiosynthesis had to be adjusted in order to minimize mechanical strain caused by heating acetone over the boiling point and to reduce the effort of maintenance of the synthesis modules, although the chemical turnover in acetone and butanone was comparable during the establishment process (see Table 1). For this reason, the solvent acetone was exchanged by butanone, which additionally enabled the omission of the cooling during the [^11^C]CH_3_OTf trapping step due to its higher boiling point. The trapping efficiency of [^11^C]CH_3_OTf in acetone and butanone was approximately 80% (non-decay corrected), in accordance to previously published data (Pichler et al. 2018b). Afterwards, the reaction solution was heated to 110 °C for 2 min, cooled to ≤35 °C and quenched with water. Semi-preparative radio-HPLC (Fig. 3) revealed the presence of the following radioactive peaks: [^11^C]CH_3_I and [^11^C]CH_3_OH (from the reaction of [^11^C]CH_3_OTf and water) at early retention times between 3 and 6 min, followed by a minor unknown side product at 8 min and the desired product fraction at approximately 12 min. In contrast to a previous study (Caillé et al. 2018) the semi-preparative HPLC column was changed from a Waters Symmetry® C18 7.8 × 300 mm, 7 μm column to a SUPELCOSIL LC-ABZ+ column. After the semi-preparative HPLC purification (Fig. 3), 3.9 ± 2.0 GBq of product was isolated, which corresponded to a radiochemical yield of 13 ± 7% (based on the starting activity of [^11^C]CO_2_, decay-corrected) or 23 ± 11% (based on trapped [^11^C]CH_3_I, decay-corrected).

### Molar activity and quality control

The molar activity of the final formulated product was 132 ± 164 GBq/μmol (range: 5–659 GBq/μmol). The molar activity was calculated according to the recently published nomenclature and harmonization guidelines (Coenen et al. 2017). The calibration curve as well as the limits of quantification and detection were determined according to ICH (International Council for Harmonisation) guidelines (see Fig. 4) (Balaram et al. 2011). If the concentration of metoclopramide was below the limit of detection (LOD), the value was set to the LOD of 0.2 μg/mL for molar activity calculation. Hence, the molar activity was presumably even better than stated in 11 out of 94 production runs, due to hitting the detection limits of the analytical method.

The quality control parameters are listed in Table 2. The parameter radiochemical purity, radionuclidic purity (γ spectrum), half-life, residual solvents as well as the physiological parameters pH and osmolality were measured before release of the product. Sterility and endotoxin testing were performed after decay of the radioactivity. All parameters were in accordance with the recommendations of the European Pharmacopoeia as applied for other ^11^C-radiotracers (European Directorate for the Quality of Medicines (EDQM) 2016).

**Table 2:** Quality control parameters for all released batches (*n* = 81) of [^11^C]metoclopramide according to the recommendations of the European Pharmacopoeia for ^11^C-radiotracers

Quality control parameter	Release criteria	Quality control
Radiochemical purity	> 95%	99 ± 1%
Molar activity	no release criteria	132 ± 164 GBq/μmol
Half-life	20.3 ± 2.0 min	19.4 ± 0.8 min
Radionuclidic purity (y-line)	430–520 keV	505 ± 15 keV
Residual solvents	< 410 ppm per solvent	< 410 ppm butanone
		< 410 ppm acetonitrile
	< 10% ethanol	< 10% ethanol
pH	4.5–8.5	7.5 ± 0.2
Osmolality	200–400 mosm/kg	316 ± 37 mosm/kg
Sterility	pass	pass
Endotoxins	Pass	pass

### Differences and similarities of TRACERlab™ FX C Pro and TRACERlab™ FX2 C

In general, the productions with the TRACERlab™ FX C Pro afforded significantly lower molar activities (62 ± 80 GBq/μmol) than the newly installed TRACERlab™ FX2 C (299 ± 184 GBq/μmol). This considerable difference in molar activity can presumably be explained by the long usage of the TRACERlab™ FX C Pro leading to carbon deposits. A detailed analysis of potential sources of carbon load in synthesizers has been published by our group recently (Pichler et al. 2018b). The final radioactivity amount of [^11^C]metoclopramide at the end of synthesis was slightly higher for the TRACERlab™ FX C Pro than for the TRACERlab™ FX2 C (4.2 ± 1.9 GBq versus 3.4 ± 2.2 GBq, not significant).

The synthetic program of [^11^C]metoclopramide of the TRACERlab™ FX C Pro had to be adjusted for the TRACERlab™ FX2 C to implement the synthesis of [^11^C]metoclopramide. The main difference between the modules is the changed installation of the silver triflate column (Fig. 2). For the TRACERlab™ FX C Pro, the silver triflate column has to be inserted before the synthesis in a horizontal position. Here, the main disadvantage lies in the recurrent manual removal and therefore opening of the column which may lead to the introduction of humidity. On the other hand, the exchange of the connecting tubes is easy and fast. For the TRACERlab™ FX2 C, the silver triflate column has a smaller column diameter, and the column is inserted in a vertical position. Here, the silver triflate column can be automatically by-passed and therefore the main disadvantage of the TRACERlab™ FX C Pro was overcome. However, a disadvantage of the TRACERlab™ FX2 C is the inaccessibility of the connecting tubing between the [^11^C]CH_3_I trap and the silver triflate column. Additionally, the heating and cooling of the silver triflate column has to take place in a mode where the valves are closed and therefore the column may be pressurized.

### Analysis of pitfalls of the [^11^C] metoclopramide production

**Fig. 6:**
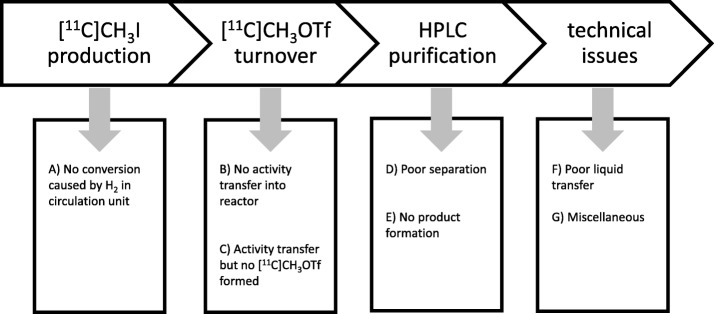
Scheme of the pitfalls of [^11^C]metoclopramide synthesis

Overall synthesis success rate was 93% combined for both modules (*n* = 94). The identified problems are depicted in Fig. 6. The first step with potential failure was the [^11^C]CH_3_I production, for which in some cases no conversion of [^11^C]CH_4_ into [^11^]CH_3_I was observed. A higher failure rate was due to insufficient transformation of [^11^C]CH_3_I into the more reactive intermediate [^11^C]CH_3_OTf. In these cases activity was either trapped on the silver triflate column or the activity was successfully transferred to the reactor, but no reaction took place due to insufficient amounts of [^11^C]CH_3_OTf. During the semi-preparative HPLC purification step two possible failure scenarios occurred: firstly, there was a poor separation between the side product and the [^11^C]metoclopramide fraction leading to an impure product and subsequently inhibiting product release. Secondly, there was no product formation at all, which may be attributed to insufficient conversion of [^11^C]CH_3_I into [^11^C]CH_3_OTf or resulting from traces of decomposed butanone. A test synthesis using [^11^C]CH_3_I as reactive synthon proved the necessity of [^11^C]CH_3_OTf for the preparation of [^11^C]metoclopramide (see Table 1). All other synthesis failures were attributable to technical issues like clogging of tubings or valves.

### Pitfalls of [^11^C]CH_3_I production

**Fig. 7:**
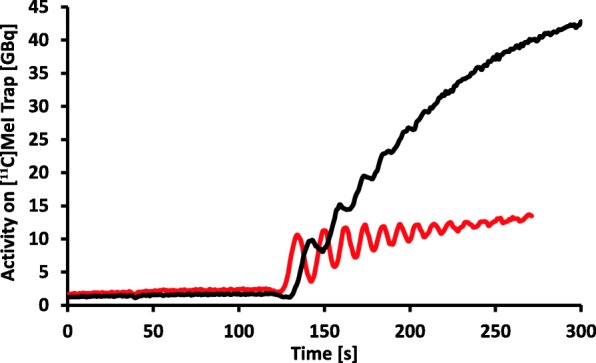
Progression of the turnover of [^11^C]CH_4_ to [^11^C]CH_3_I and trapping of the activity on the methyl iodide trap, for a successful reaction (black) and failed reaction caused by presence of hydrogen (red) in the circulation unit of the GE TRACERlab™ FX C Pro synthesizer

In general, the gas-phase [^11^C]CH_3_I production was very stable and reproducible as described before (Pichler et al. 2018b). However, a potential source of failure in the [^11^C]CH_3_I production could be identified in the course of the evaluation of [^11^C]metoclopramide. In these cases, the characteristic pattern of the re-circulation of [^11^C]CH_4_ and trapping of [^11^C]CH_3_I was not observed. Instead, the radioactivity was passing through the [^11^C]CH_3_I trap without any trapping (see Fig. 7). Hence, this failure in [^11^C]CH_3_I production was therefore visible within the first few seconds of the re-circulation process. Once the failure in trapping was noticed, the circulation unit could be flushed with helium and the synthesis could be immediately re-started, as the precursor solution was not affected. In these rare cases, the second run was always successful.

The cause for this failure could be confirmed and the erroneous circulation pattern could be reproduced when the whole circuit unit was flushed with hydrogen (100 mL/min for 2–3 min) instead of helium prior to the [^11^C]CH_3_I production. The introduction of hydrogen into the unit is only explicable, if a valve is leaking or a faulty circuit happens during the conditioning of the CO_2_ trap. Other (non-commercial) gas-phase [^11^C]CH_3_I production units are not trapping the formed [^11^C]CH_4_ on a cooled methane trap (personal communication, see also methane trap in Fig. 2) and therefore the produced [^11^C]CH_4_ is not separated from remaining [^11^C]CO_2_ and unreacted H_2_. As the production of [^11^C]CH_3_I in these production units is working properly, we assume that this problem may only occur in presence of high amounts of hydrogen.

### Validation of [^11^C]CH_3_OTf production

**Fig. 8:**
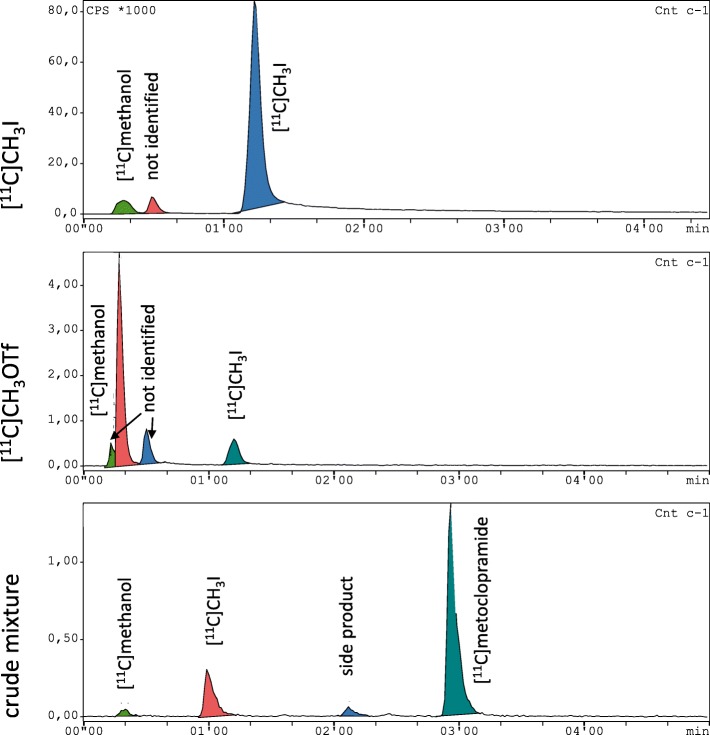
Radio-HPLC analysis of [^11^C]CH_3_I, [^11^C]CH_3_OTf and crude reaction mixture. The individual peaks were identified by co-injection of commercially available unlabelled reference compounds. The analyzed [^11^C]CH_3_OTf batch contained approximately 13% of [^11^C]CH_3_I due to incomplete conversion of [^11^C]CH_3_I into [^11^C]CH_3_OTf. [^11^C] methanol originates from the reaction of [^11^C]CH_3_OTf with water. A similar phenomenon was seen for the crude reaction mixture

Overall, the [^11^C]CH_3_OTf production was more prone to failure than the [^11^C]CH_3_I production and was the main source of synthesis failure. With the set-up of the TRACERlab™ FX2 C module, two different unwanted scenarios were observed. In the first scenario, the [^11^C]CH_3_I was either almost completely trapped on the silver triflate column or insufficiently transferred from the silver triflate column into the reactor. In the second scenario, a very good transfer of the radioactivity into the reactor was observed, but there was no conversion of [^11^C]CH_3_I into [^11^C]CH_3_OTf. The trapping of the [^11^C]CH_3_I on the silver triflate column primarily occurred in our facility, when the silver triflate column was exposed to humidity. However, the main source of failure was the insufficient transformation of [^11^C]CH_3_I into [^11^C]CH_3_OTf. Consequently, the silver triflate column must be checked and replaced regularly in order to guarantee a stable process. The exchange of the silver triflate column was performed every 15th – 20th synthesis for the GE TRACERlab™ FX C Pro. The successful transformation of [^11^C]CH_3_I into [^11^C]CH_3_OTf was indirectly detected by analytical HPLC analysis of the crude reaction mixture. Although [^11^C]CH_3_I reacts with water of the mobile phase to [^11^C]CH_3_OH, a certain amount of non-reacted [^11^C]CH_3_I was detectable (see Fig. 8). [^11^C]CH_3_OTf, on the other hand, reacts very quickly with water, so that no [^11^C]CH_3_OTf peak is detectable but a pronounced [^11^C]CH_3_OH peak. For failure analysis after the synthesis, the injection of the crude reaction mixture into analytical HPLC allows the following conclusions: (i) a detectable [^11^C]CH_3_I peak indicated an insufficient conversion to [^11^C]CH_3_OTf, which considerably lowers the radiochemical yield; (ii) if only the [^11^C]CH_3_OH peak was present, the reaction of [^11^C]CH_3_I to [^11^C]CH_3_OTf took place, so that other factors, like insufficiently inert atmosphere, among others, were responsible for low product conversion.

### Separation efficiency of semi-preparative HPLC and other technical issues

**Fig. 9:**
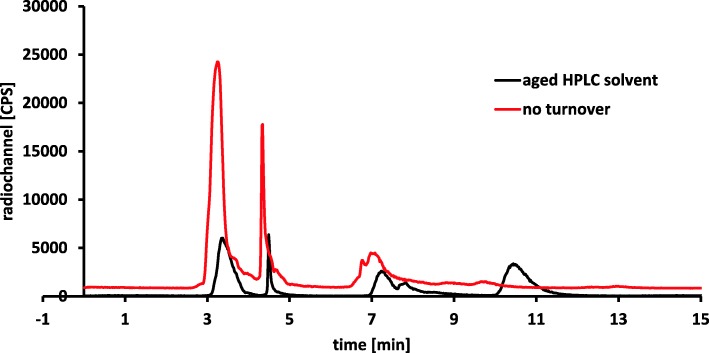
Insufficient semi-preparative RP-HPLC chromatograms caused by aged HPLC solvent (black) or decomposed butanone (red)

A poor semi-preparative chromatogram was present, when either there was an insufficient separation of product and side product or there was no product formation at all. For an efficient purification of [^11^C] metoclopramide it was necessary to prepare the mobile phase freshly at least once per week. Otherwise the tailing of the [^11^C]metoclopramide peak enhanced (Fig. 9), as well as the amount of the side peak at around 8 min appeared to increase. Analysis of the collected semi-preparative HPLC product fraction by analytical HPLC showed that the collected peak consist of both, side product and [^11^C]metoclopramide. Exchange of the HPLC solvent restored the separation and reduced the peak at approximately 8 min.

A reduced or no formation of the product was either due to poor [^11^C]CH_3_OTf formation but also caused by decomposition of the butanone after long time usage (Fig. 9). Acetone is known to undergo aldol reactions when stored on molecular sieves (JoVE Science Education Database 2019). However, GC and ^1^H-NMR did not show any aldol reaction product in amounts higher than 0.01% (GC analysis) or at all (^1^H-NMR) in butanone, which was stored over molecular sieves for more than 6 months. Still, the olfactory differences between the bottle of butanone stored over molecular sieves and a bottle of butanone without molecular sieves suggests that butanone underwent molecular sieve-catalyzed reactions. This small amount of formed aldol product might still be enough to interfere with the radiolabeling via [^11^C]CH_3_OTf. Therefore, a regular exchange of butanone at least every 3 months is recommended.

## Conclusion

[^11^C]Metoclopramide was reliably synthesized on both, a GE TRACERlab™ FX C Pro and GE TRACERlab™ FX2 C synthesis module, for more than 100 synthesis in good yields and high molar activities. The quality of the final product was in accordance with the recommendations of the European Pharmacopoeia for other ^11^C-radiotracers. The main technical problems were identified, and individual solutions were developed in order to keep synthesis failures at a minimum.

## Data Availability

Please contact corresponding author for data request.

## Abbreviations

PET: positron emission tomography
P-gp: P-glycoprotein
RP-HPLC: reversed phase-high performance liquid chromatography
